# Efficacy of routine contrast echocardiography for the detection of left ventricular thrombus in patients with anterior ST-elevation myocardial infarction

**DOI:** 10.1186/s44348-024-00041-2

**Published:** 2024-11-21

**Authors:** Hui-Jeong Hwang, Il Suk Sohn

**Affiliations:** Department of Cardiology, Kyung Hee University Hospital at Gangdong, Kyung Hee University College of Medicine, Seoul, Republic of Korea

The incidence of left ventricular (LV) thrombus has significantly decreased since primary percutaneous coronary intervention (PCI) became widely practiced as a first-line therapy [1]. A meta-analysis of 16 studies reported during the primary PCI era (*n* = 3,447) demonstrated that LV thrombus following anterior ST-segment elevation myocardial infarction (STEMI) was observed to be in the range of 9.1% (95% confidence interval, 6.6%–11.6%) [2]. However, the detection rate of LV thrombus based on conventional echocardiography may be underestimated, expecially in cases of small or mural LV thrombus or in poor acoustic windows [1, 3, 4]. Furthermore, delayed diagnosis of LV thrombus may lead to embolic events such as stroke; therefore, its early detection is crucial [5].

Contrast echocardiography, using contrasts agents for LV opacification, helps differentiate ultrasound artifacts such as near-field clutter (Fig. 1A, Additional file 1) or LV trabeculation (Fig. 1B, Additional file 2) from LV thrombus. Moreover, it can be used to exclude LV thrombus in patients with poor echocardiographic window (Fig. 1C, D, Additional files 3, 4). In a recent issue of the *Journal of Cardiovascular Imaging*, Correia et al. [6] published a study titled “Left ventricular thrombus routine screening with contrast echocardiography in patients with anterior ST-elevation myocardial infarction: is it worth it?” The study investigated whether contrast echocardiography is more effective in dectecing LV thrombus compared to conventional echocardiography. In their study, the detection rate of LV thrombus was higher in the group that underwent contrast echocardiography (25%) compared to the group that underwent conventional echocardiography (14%), but the difference was not signficant (*P* = 0.24). Conversely, the detection rate of anterior or apical LV aneurysms was significantly higher in the group that underwent contrast echocardiography (47% vs. 22%, *P* = 0.03). Despite the higher detection rate of LV thrombus in this study compared to that in previous studies [2], the failure to demonstrate significant efficacy of contrast echocardiography may be due to the small sample size of the study (32 in the contrast echocardiography group; 36 in the conventional echocardiography group). Of note, the use of contrast echocardiography improved the detection rate of anterior or apical LV aneurysms, which are risk factors for subsequent LV thrombus. Therefore, this study demonstrates that routine use of contrast echocardiography is useful, at least for screening patients at risk for LV thrombosis.

**Fig. 1:**
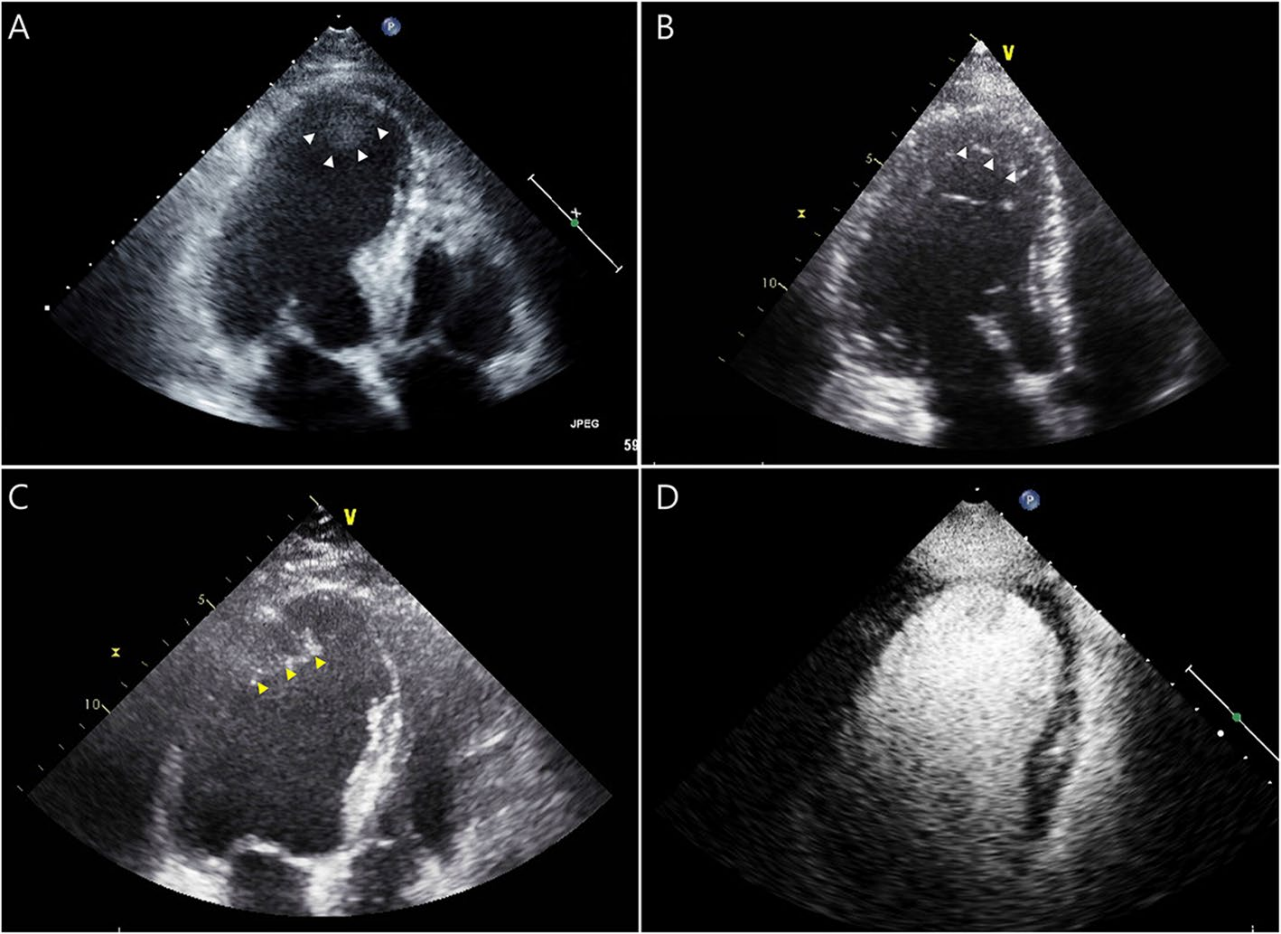
Several examples of suspected left ventricular thrombus. **A** Apical four-chamber view on conventional echocardiography, showing near-field clutter (white arrow). **B** Conventional echocardiography displaying left ventricular trabeculation (white arrow). **C** Suspicion of left ventricular thrombus (yellow arrow) on conventional echocardiography. **D** Contrast echocardiography demonstrating the exclusion of left ventricular thrombus

The detection rate of LV thrombus on cardiac magnetic resonance in patients with anterior STEMI may be superior to that on conventional echocardiography [7]. However, cardiac magnetic resonance has several clinical limitations, such as the requirement for adequate creatinine clearance, the need for patients to maintain a stable condition, a long imaging time, and the necessity for high-technology equipment. On the contrary, contrast echocardiography is easily accessible and has minimal effect on the clincial condition of patients. Therefore, contrast echocardiography may be a suitable option for better detection of LV thrombus compared to conventional echocardiography in patients with anterior STEMI, particularly when dealing with a poor echocardiographic window.

## Supplementary Information

Additional file 1. Supplementary Video 1.

Additional file 2. Supplementary Video 2.

Additional file 3. Supplementary Video 3.

Additional file 4. Supplementary Video 4.

## Data Availability

Not applicable.

## Abbreviations

LV: Left ventricular
PCI: Percutaneous coronary intervention
STEMI: ST-segment elevation myocardial infarction
